# Functional Insights into the Roles of Hormones in the *Dendrobium officinale-Tulasnella* sp. Germinated Seed Symbiotic Association

**DOI:** 10.3390/ijms19113484

**Published:** 2018-11-06

**Authors:** Tao Wang, Zheng Song, Xiaojing Wang, Lijun Xu, Qiwu Sun, Lubin Li

**Affiliations:** 1 State Key Laboratory of Forest Genetics and Tree Breeding, Key Laboratory of Silviculture of the State Forestry Administration, Research Institute of Forestry, Chinese Academy of Forestry, Beijing 100091, China; wangtao5757@126.com (T.W.); songzheng566@163.com (Z.S.); wangxjwork@163.com (X.W.); xulijun19935@126.com (L.X.); 2 Beijing Botanical Garden, Beijing 100093, China

**Keywords:** *Dendrobium officinale*, *Tulasnella* sp., symbiosis, endogenous hormones

## Abstract

*Dendrobium* is one of the largest genera in the Orchidaceae, and *D. officinale* is used in traditional medicine, particularly in China. *D. officinale* seeds are minute and contain limited energy reserves, and colonization by a compatible fungus is essential for germination under natural conditions. When the orchid mycorrhizal fungi (OMF) initiates symbiotic interactions with germination-driven orchid seeds, phytohormones from the orchid or the fungus play key roles, but the details of the possible biochemical pathways are still poorly understood. In the present study, we established a symbiotic system between *D. officinale* and *Tulasnella* sp. for seed germination. RNA-Seq was used to construct libraries of symbiotic-germinated seeds (DoTc), asymbiotic-germinated seeds (Do), and free-living OMF (Tc) to investigate the expression profiles of biosynthesis and metabolism pathway genes for three classes of endogenous hormones: JA (jasmonic acid), ABA (abscisic acid) and SLs (strigolactones), in *D. officinale* seeds and OMF under symbiotic and asymbiotic conditions. Low concentrations of endogenous JA, ABA, or SLs were detected in the *D. officinale-Tulasnella* symbiont compared with the asymbiotic tissues. Gene annotation results suggest that the expression of DEGs (differentially expressed genes) related to JA and ABA biosynthesis from *D. officinale* were down-regulated, while most of the key DEGs related to SL biosynthesis from *D. officinale* were up-regulated in the symbiotic germinated seeds compared with the asymbiotic germinated seeds. Moreover, in the OMF, we found a significantly up-regulated differential expression of the JA and ABA biosynthesis-related genes in the symbiotic interaction, with the opposite expression trends to those found in *Dendrobium*. This indicates that *Dendrobium* seed symbiotic germination may be stimulated by the apparent involvement of the OMF in the production of hormones, and relatively low concentrations of endogenous JA, ABA, or SLs might be maintained to promote the growth of the *D. officinale-Tulasnella* symbiotic protocorm-like body. These results will increase our understanding of the possible roles played by endogenous hormones in the regulation of the orchid-fungus symbiosis.

## 1. Introduction

In the majority of orchid species, seed germination is largely dependent on mycorrhizal fungi under natural conditions because of the lack of nutritional reserves [1]. Symbiotic seed germination, which is essential for orchid propagation and reintroduction, can be used for the conservation of the *Dendrobium* species in their natural habitats [2]. The process of orchid seed germination under natural conditions is complex and unique, and involves various physiological and biochemical processes such as colonization, plant growth stimulation, and the plant response to the fungus [1]. Although successes in orchid seed germination are regularly reported in the literature, it is important to note that when comparing the asymbiotic and symbiotic germination of the same species, the rates of orchid seed germination, development of the protocorms, and growth is usually better in the latter [3,4]. Many studies have shown that orchid mycorrhizal fungi (OMF) promote metabolic processes in orchid seeds [4,5]. Although orchid seeds contain very low nutrient reserves, recent analyses of the unique properties of protocorms have shown that during symbiotic seed germination, the expression of putative genes involved in metabolism, transcriptional regulation, and signal transduction pathways is upregulated [5,6,7]. Miura et al. (2018) examined an orchid-mycorrhizal symbiosis by analyzing the transcriptome of *Bletilla striata* associated with *Tulasnella* sp. at an early developmental stage, and the essential genes required for the establishment of a mutualistic relationship with AM fungi and/or rhizobia in most terrestrial plants were identified from *B. striata* [8]. These studies indicate that the symbiotic association formed from the germinated seed is able to interact with mycorrhizal fungi.

When mycorrhizal fungi undergo symbiotic interactions with orchids, phytohormones produced by the orchid and/or the microbial partners could play key roles [9,10,11]. The symbiosis between *Cymbidum goeringii* and a *Rhizoctonia*-like mycorrhizal fungi causes the release of hormones, which can promote the growth of *C. goeringii* seedlings [9]. The results of comparative transcriptomic and proteomic analyses suggested that an ABA receptor protein, PYR1, showed a significantly higher expression during the early symbiotic germination stages (when the embryo swells, enlarges, and emerges from the seed coat) in *D. officinale* [10]; the developmental stages are defined according to Stewart et al. [12]. The role of JA signaling is well known in plant–pathogen interactions [11]. A recent study also detected higher levels of lipoxygenase in *Oncidium sphacelatum* at the green protocorm stage of seed development with *Ceratobasidium* sp., and this could possibly result in an enhanced JA biosynthesis in the green protocorms with the fungal symbiont [6]. These studies indicate that the functions of the growth-inhibiting hormones ABA and JA could be important for symbiotic seed germination and growth of *Dendrobium*.

In higher plants, ABA is derived from the oxidative cleavage of carotenoids. Isopentenyl diphosphate (IPP) formed in the 1-deoxy-d-xylulose-5-phosphate (DXP) pathway is converted to geranylgeranyl pyrophosphate (GGPP), from which carotenoids are synthesized [13,14]. The carotenoid β-carotene is hydroxylated to form the xanthophyll zeaxanthin, which is further converted into ABA by a series of enzymes, such as zeaxanthin epoxidase (ZXE) and short-chain dehydrogenase/reductase (SDR). It has been reported that the symbiotic fungi, including OMF, can also produce compounds that are similar to the plant hormome ABA, although studies have shown that the ABA biosynthetic pathways seem to differ between higher plants and fungi [15]. Fungi synthesize ABA via a direct pathway from isopentenyl diphosphate (IDP) and farnesyl diphosphate (FDP), which are derived from the mevalonic acid (MVA) pathway [16,17]. After a series of cyclization, isomerization, and oxidization reactions, ABA is synthesized from farnesyl pyrophosphate (FPP). Labeling experiments have been performed to characterize the ABA biosynthesis pathway in *Botrytis cinerea, Cercospora rosicolla*, and some other species of fungi [18,19,20]. Previous studies have shown that 1′,4′-*trans*-diol-ABA is the main precursor of ABA in *B. cinerea* and that ∆*BcABA4* transformants accumulate 1′,4′-*trans*-diol-ABA [17]. The *BcABA4* gene encodes a putative SDR (short-chain-type dehydrogenase/reductase) which is involved in the ABA biosynthesis in *Arabidopsis* [16].

The JA biosynthesis pathways of plants and OMF are more similar. JA synthesis is initiated in the plastid by the oxygenation of linoleic or α-linolenic acid by lipoxygenases (LOXs) to form (13*S*)-hydroperoxy linoleic or α-linolenic acid. This is then followed by the first committed step of JA biosynthesis catalyzed by allene oxide synthase (AOS) that converts 13-HPOT to a highly reactive allene oxide, which is converted to *cis*-(+)-12-oxophytodienoic acid by allene oxide cyclase (AOC). *cis*-(+)-OPDA is then transported to the peroxisome, where 12-oxophytodienoate reductase 3 (OPR3) and three rounds of β-oxidation convert it to JA [21].

Previous studies have shown that plants generally produce multiple strigolactone (SL) species which are released from the roots and induce germination in seeds of the *Striga* and *Phelipanche* species in the soil [22,23]. SLs or their biosynthetic precursors act as plant hormones to inhibit shoot branching in plants [24], which was discovered in studies using a series of enhanced shoot branching mutants [22,23,24,25]. SLs also function as host recognition signals for AM (arbuscular mycorrhizal) fungi, as well as branching factors in AM fungi [22,23]. A hypothetical SL-biosynthetic pathway was initially proposed, with β-carotene as a substrate for carotenoid-cleavage dioxygenase enzymes (CCD7 and CCD8, which act sequentially in the pathway). Further investigation of the *MAX1* gene, which encodes a cytochrome P450 in the CYP711A1 clade, acting downstream to CCD8 and of its orthologs in *Arabidopsis* and rice revealed that this gene is expressed in all vascular tissues, and functions only in the late steps of SL synthesis [26]. D14/DAD2 (a probable strigolactone esterase) may be a putative SL receptor involved in SL signaling pathway regulation. Although it has been hypothesized that SL phytohormones have important functions during plant-fungus interactions and symbioses [23], little is known about the role of SLs in the symbiotic associations between orchids and OMF.

These phytohormones are well known to control plant development and to trigger important signaling events [15]. Moreover, plant hormones produced by mycorrhizal fungi have been shown to improve the growth of medicinal orchids [4]. In addition, the possible biosynthesis pathways for plant and fungal hormones in the orchid–fungal symbiosis are poorly understood at present. The manner in which these molecules favor the invasion of plant tissues and the development of fungi inside plant tissues is also largely unknown. To this end, we performed comparative transcriptomic and hormone profiling between symbiotically and asymbiotically germinated *D. officinale* seeds in an effort to gain possible functional insights into the biosynthesis and metabolism of ABA, JA, and SLs in the regulatory module in the *Dendrobium*–*Tulasnella* symbiosis. We also predicted some unigenes involved in phytohormone production by the symbiotic fungus *Tulasnella* sp., and these genes had different expression profiles in symbiotic and asymbiotic germinated seeds.

## 2. Results

### 2.1. Detecting Mycorrhizal Fungus Colonization in Germinated Seeds of D. officinale

**Figure 1:**
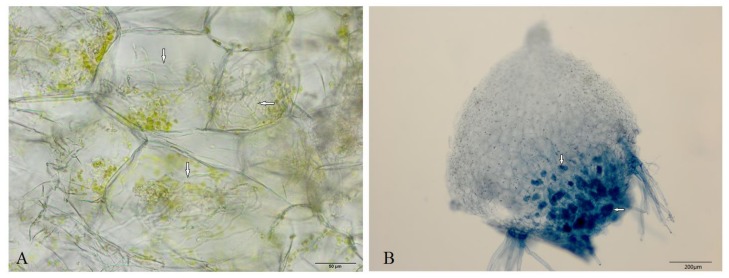
The morphological observation of intracellular hyphae in the symbiotic germinated seeds of *D. officinale*. The microscopic observation of intracellular hyphae in a free-hand section (**A**) of germinated seeds were stained blue following Trypan blue staining (**B**). The arrows indicate the symbiotic mycelia.

Both morphological observation and Trypan blue staining, which showed that the intracellular fungal hyphae stained blue in the symbiotic germinated seeds (DoTc; Figure 1), indicated that a symbiotic relationship had been established between *D. officinale* germinated seeds and *Tulasnella* sp., while in the group of seeds germinated without fungi with a low germination rate (0.1%), we did not find hyphae, and these were therefore defined as asymbiotic germinated seeds (Do).

### 2.2. Transcriptome Sequence Assembly and Annotation.

To globally characterize the transcriptome of *D. officinale* and its *Tulasnella* endosymbiont with enhanced sequence coverage, nine cDNA libraries were constructed using RNA extracted from DoTc, Do, and free-living *Tulasnella* mycelium (Tc). In total, 667,270,886 Illumina paired-end raw reads were generated. The raw reads are available in the NCBI SRA database under the accession number SRP136098. After discarding the adaptor and low-quality sequences, we obtained 642,119,290 clean reads (accounting for 96 Gb of DNA sequence). The percentages of clean reads among the raw tags (Q20) ranged from 95.52% to 96.11% (Table S1). The cleaned reads of *D. officinale* from the DoTc and Do libraries were then mapped to the *D. officinale* genome database [27] with 45.7–50.27% and 60.62–61.04% of the reads in the DoTc and Do libraries, respectively, mapping to the genome (Table S2). The novel genes annotated in *Dendrobium officinale* are shown in Table S3.

The de novo assembly of the clean reads was performed and resulted in 50,597 unigenes that ranged from 201 to 12,137 bp in length, with an N50 length of 1488 bp in the *Tulasnella* transcriptome. The size distribution of the transcripts and unigenes in *Tulasnella* is shown in Table S4. The transcriptome assembly produced a substantial number of large unigenes: 15,254 unigenes were >1000-bp in length (Table S4). All assembled unigenes (Table S5) were annotated using BLASTX searches against the NR, NT, KO, SwissProt, PFAM, GO, and KOG databases (Table S6). In total, there were 1637 (3.23%) unigenes that were annotated in all databases, and 35,616 (79.39%) that were annotated in at least one database (Table S6), providing significant BLAST results. Among the annotated unigenes, 23,758 (46.95%) showed significant similarity to known proteins in the Nr database, 23,532 unigenes (46.50%) in the GO database, and 9105 unigenes (17.99%) in the KOG database (Table S6).

### 2.3. Differentially Expressed Genes in Symbiotic and Asymbiotic Germinated Seeds and Mycorrhizal Fungi

**Figure 2:**
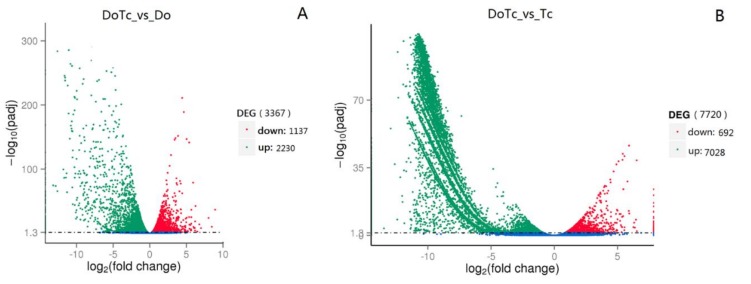
The distribution of differentially expressed genes (DEGs) in symbiotic and asymbiotic germinated seeds. (**A**) DEGs originating from *D. officinale* in the symbiotic germinated seeds (DoTc vs. Do), and (**B**) DEGs originating from OMF (orchid mycorrhizal fungi) in the symbiotic germinated seeds (DoTc vs. Tc). Genes in which the relative level of expression was down-regulated are shown in red, and those that were up-regulated are shown in green.

To compare the differences in gene expression between the symbiotic germinated seeds, asymbiotic germinated seeds, and mycorrhizal fungi, we compared the transcriptome profiles between the symbiotic and asymbiotic germinated seeds (DoTc vs. Do) and symbiotic and asymbiotic mycorrhizal fungi (DoTc vs. Tc). A total of 3367 DEGs (differentially expressed genes) were obtained that included 2230 up-regulated and 1137 down-regulated genes in the DoTc vs. Do comparison (Figure 2A and Table S7), and 7720 differentially expressed unigenes that included 7028 up-regulated and 692 down-regulated unigenes in the DoTc vs. Tc comparison (Figure 2B and Table S8).

The annotation results suggest that orchid seed germination stimulated by the fungal association apparently involves plant hormone signal transduction, plant-pathogen interaction, and metabolic pathways from *D. officinale* that were obviously up-regulated (Figure S1) in the symbiotic germinated seeds (DoTc vs. Do). However, in the mycorrhizal fungus, the expression of genes for proteins implicated in the ribosome, proteasome biosynthesis, and protein processing in the endoplasmic reticulum were found in the symbiotic interaction (Figure S1, DoTc vs. Tc).

It is well known that plant hormones have important functions during plant-fungal interactions. In the present study, we found that DEGs related to hormone pathways show a significant differential expression (Figure S1A); we thus sought to gain possible functional insights into the biosynthesis and metabolism of three types of plant hormones—JA, ABA, and SL—in the regulatory module of the *Dendrobium*–*Tulasnella* symbiosis. In the ABA biosynthesis pathway, the expression of *ZXEs* and *SDRs* in *D. officinale* was significantly down-regulated in the symbiotic germinated seeds compared with the asymbiotic germinated seeds. However, the possible ABA biosynthesis-related short-chain type dehydrogenase/reductase-encoding gene *BcABA4* [16] and the P450 monooxygenase-encoding genes *BcABA1/2* [17] were significantly up-regulated in the symbiotic germinated seeds compared with free-living OMF (Table 1). Moreover, the JA biosynthesis pathway-related genes from the plant and OMF showed similar expression profiles in the symbiotic seeds, but with opposite trends. We have observed that some JA biosynthesis-related enzyme-encoding genes, such as LOX, AOS, OPR, and 3-ketoacyl-CoA thiolase (KAT) are down-regulated in the symbiotic germinated seeds relative to their expression in the asymbiotic germinated seeds, while the key genes in the *Tulasnella* JA biosynthesis pathway showed an up-regulated expression in the symbiotic germinated seeds. In the SL biosynthesis pathway, the isomerase-encoding gene *D27*, two carotenoid-cleavage dioxygenases *CCD7* and *CCD8*, and the *CYP450* monooxygenase gene *MAX1* were all up-regulated in the symbiotic germination seeds. In addition, five *D14*/*DAD2* genes were also found to be up-regulated in the *D. officinale-Tulasnella* symbiotic germination seeds.

### 2.4. Expression of DEGs Related to ABA, JA, and SL Hormone Biosynthesis Detected by qRT-PCR

**Figure 3:**
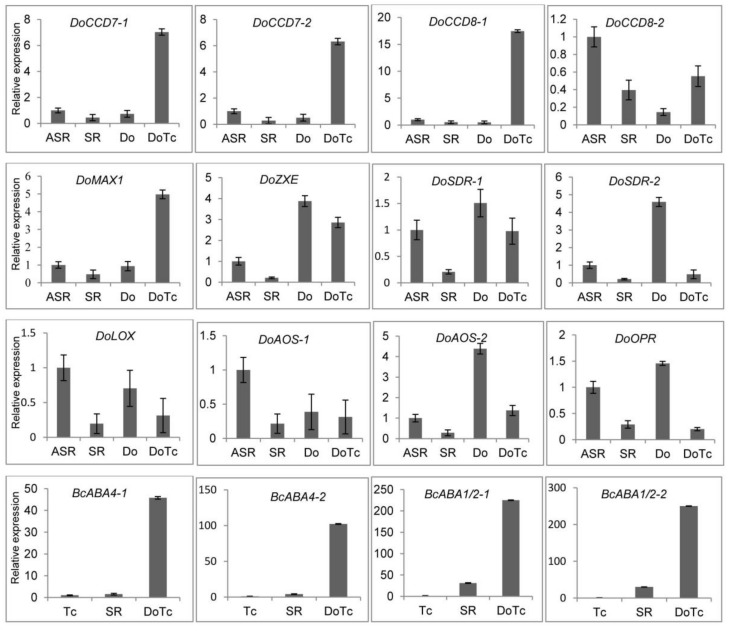
The expression levels of hormone biosynthesis-related genes determined by qRT-PCR. DoTc: symbiotic germinated seeds; Do: asymbiotic germinated seeds; SR: symbiotic roots of *D. officinale* seedlings; ASR: asymbiotic roots of *D. officinale* seedlings; Tc: free-living *Tulasnella* mycelium. Each data point represents the mean of three biological replicates and three experimental replicates. Bars represent the standard error of the mean, SE (*n* = 9).

In the symbiotic and asymbiotic germinated seed samples, we successfully detected the expression of key genes related to ABA, JA, and SL biosynthesis, and the results are shown in Figure 3. Two *CCD7* and *CCD8* genes and the *MAX1* gene, which are key genes in the *D. officinale* SL biosynthesis pathway, all showed up-regulated expression in the symbiotic germinated seed (DoTc) samples, based on expression profiles determined from transcriptome sequencing. In the symbiotic roots of *D. officinale* seedlings (SR), these genes were all down-regulated compared to the control asymbiotic root samples (ASR), which showed expression trends that were opposite to the SGS samples, with the exception of *CCD8-2*. In the ABA and JA biosynthesis pathways, the expression of the *ZXE*, *SDR-1* and *SDR-2*, *LOX*, *AOS-1* and *AOS-2*, and *OPR* genes from *D. officinale* was significantly down-regulated in the SGS and SR samples compared to one or both of the asymbiotic control samples (Do and ASR). Moreover, we also assayed the differential expression of four *BcABA* genes (*BcABA4-1*, *BcABA4-2*, *BcABA1/2-1*, and *BcABA1/2-2*) which could be involved in ABA biosynthesis in the OMF *Tulasnella* sp. in the SGS and SR samples and free-living *Tulasnella* mycelium (Tc). We found that the expression in the SGS and SR samples was significantly up-regulated compared with that in the Tc samples, and the expression of these four genes in the SGS samples was also based on the expression trends from the transcriptome sequencing and was more significantly upregulated than in the SR samples.

### 2.5. Assays of ABA, JA, and SL Contents

**Figure 4:**
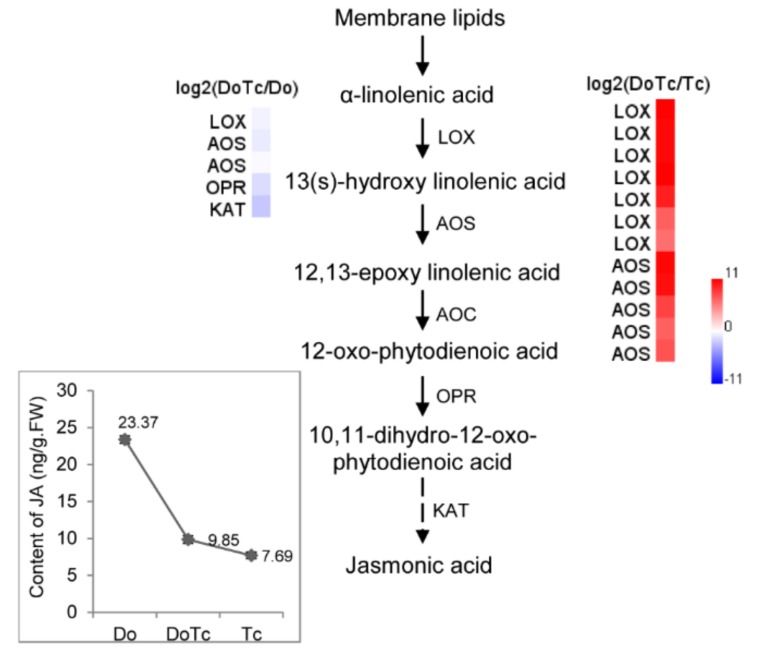
The genes involved in the biosynthesis of the oxylipin JA identified in the symbiotic germinated seeds of *D. officinale* in association with a *Tulasnella* sp. isolate. LOX: Linoleate 13S-lipoxygenase; AOS: Allene oxide synthase; AOC: Allene oxide cyclase; OPR: 12-oxophytodienoate reductase; KAT: 3-ketoacyl-CoA thiolase; JMT: Jasmonate O-methyltransferase. Red and blue indicate up- and down-regulated transcripts, respectively, from the DoTc vs. Do and DoTc vs. Tc comparisons. Each data point represents the mean of six biological replicates. Bars represent the standard error of the mean, SE (*n* = 6).

**Figure 5:**
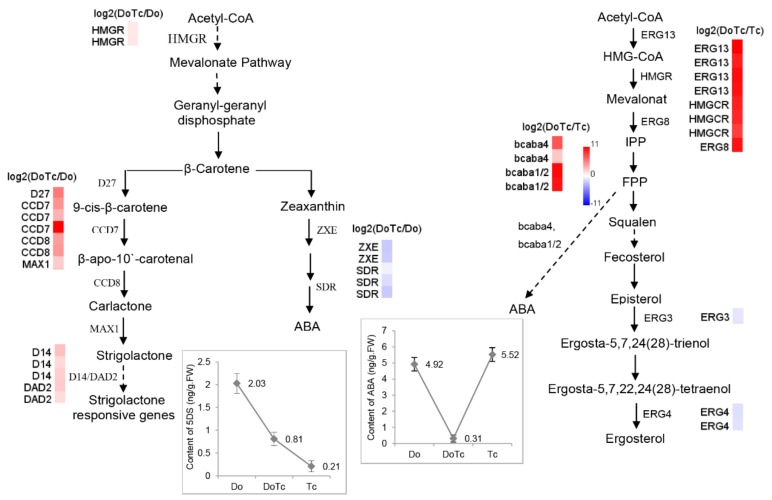
The diagram showing the proposed stigmasterol, ABA, and ergosterol biosynthesis pathways in the symbiotic germinated seeds of *D. officinale* in the symbiotic association with a *Tulasnella* sp. isolate. HMGR: Hydroxymethylglutaryl-CoA reductase; D27: β-carotene isomerase; CCD: carotenoid cleavage dioxygenase; MAX: more axillary growth; ZXE: zeaxanthin epoxidase, SDR: short-chain dehydrogenase/reductase; HMG: Hydroxymethylglutaryl; ERG13/HMGS: Hydroxymethylglutaryl-CoA synthase; ERG8: phosphomevalonate kinase; FPP: Farnesyl-Pyrophosphate; ERG4: sterol C-24 reductase. Red and blue indicate up- and down-regulated transcripts, respectively, from the DoTc vs. Do and DoTc vs. Tc comparisons. Each data point represents the mean of six biological replicates. Bars represent the standard error of the mean, SE (*n* = 6). The solid arrows represent direct relationships, and the dashed arrows represent indirect relationships.

In order to perform a deep analysis to determine their possible functions, we assayed the absolute content of ABA, JA, and 5-deoxystrigol (5-DS) in the symbiotic germinated seeds, asymbiotic germinated seeds, and free-living *Tulasnella* mycelium, corresponding to the RNA-Seq libraries DoTc, Do, and Tc, respectively. The results showed that in the symbiotic *D. officinale* germinated seeds, the JA concentration was slightly higher than in free-living *Tulasnella* mycelium and much lower than in the asymbiotic germinated seeds (Figure 4), and the ABA concentration was lower than in the two types of control samples (Figure 5). 5-DS, a precursor of SLs, is an essential index for quantifying SLs [28]. The 5-DS contents in the different tissues varied from 0.21 to 2.03 ng/g·FW); the lowest content was in the free-living *Tulasnella* mycelium, followed by the symbiotic germinated seeds, with the highest content found in the asymbiotic germinated seeds (Figure 5).

## 3. Discussion

The role of JA signaling is well known in plant–pathogen interactions, and it also possibly results in the enhanced biosynthesis of JA in the green protocorms of *Oncidium sphacelatum* in association with the fungal symbiont *Ceratobasidium* sp. [6]. We have observed that the JA concentration in the symbionts is lower than in asymbiotic germinated seeds, but it is higher than in free-living fungal hyphae of *Tulasnella* sp. (Figure 4). Moreover, the trend of changes in JA concentration seen in the symbionts and control samples is supported by the relative expression of JA biosynthesis pathway genes. We observed that some JA biosynthesis-related enzyme-encoding genes, such as *LOX*, *AOS*, *OPR*, and *KAT*, were down-regulated in the symbiotic germinated seeds relative to their expression in the asymbiotic germinated seeds, which suggested that JA, as a kind of defense-associated plant hormone, may also be involved in the regulation of *D. officinale* seed association with mycorrhizal fungi. We can speculate that the colonization of the protocorms by the *Tulasnella* fungus resulted in the downgrading of JA biosynthesis-related genes, and allowed the fungus to enter cortical cells unimpeded and set up an efficient mutualism. The key genes in the *Tulasnella* JA biosynthesis pathway showed a significantly up-regulated expression, indicating that JA produced by *Tulasnella* might accumulate to a high level in the symbiotic germinated seeds.

The putative biosynthesis network consisting of the ABA and SL biosynthesis pathways in the symbiotic germinated seeds of the *D. officinale-Tulasnella* symbiosis is shown in Figure 5. In this network, the *HMGR* gene that encodes 3-hydroxy-3-methylglutaryl-CoA reductase, which catalyzes the reduction of 3-hydroxy-3-methylglutaryl-CoA in the mevalonate pathway, showed an induced expression in the symbiotic germinated seeds. Geranylgeranyl-PP is then catalyzed to form β-carotene, which is the critical branching point for both SL and ABA, and many of the catalyzing enzyme-related genes showed different degrees of expression in the two types of germinated seeds.

Many studies have shown a negative correlation between the concentrations of endogenous ABA and rates of seed germination, which supports the notion that high concentrations of endogenous ABA in seeds may play a critical role in preventing germination in species such as wheat (*Triticum aestivum* L.) [29], cacao (*Theobroma cacao* L.) [30], and rapeseed (*Brassica rapa* L.) [31]. In orchids, it has been suggested that high concentrations of ABA occur in seeds that are difficult to germinate such as *Epipactis helleborine* [32], and *Calanthe tricarinata* [3]. Our results showed that in symbiotic germinated *D. officinale* seeds, the ABA concentration was lower than in the control, indicating that low endogenous ABA levels may have positive effects on *D. officinale* symbiotic seed germination. Previous studies have shown that in symbiotic *D. officinale* seedlings, the ABA concentration was lower than in the control [33], which is in accord with the lower ABA concentration trends observed in the symbiotic germinated seeds compared to the asymbiotic germinated seeds. In our study, the expression of *SDR* and *ZXE*, two genes in the ABA biosynthesis pathway, was significantly suppressed in the symbiotic germinated seeds. These results agree with those of a recent study in which high concentrations of exogenously applied ABA were also found to suppress seed germination in vitro [3].

In our study, the relative transcription of most of the key MVA pathway genes from *Tulasnella* was significantly up-regulated, as was the transcription of some critical ABA biosynthesis unigenes with homologs in *Botrytis*; examples are two putative *BcABA4* unigenes (Cluster-2641.12008 with 7.24-fold up-regulation and Cluster-2152.0 with 2.42-fold up-regulation) and two putative P450 monooxygenase protein CYP51 genes (*BcABA1* or *BcABA2*; Cluster-2641.19250 with 10.43-fold up-regulation and Cluster-2641.15199 with 10.01-fold up-regulation) in the symbiotic germinated seeds compared to the asymbiotic fungus *Tulasnella* sp. (Figure 5 and Table 1), indicating that symbiotic *Tulasnella* sp. might produce more activated ABA than the control. These results are consistent with previous reports showing that ABA affects mycelium growth [11]. In *Ceratocystis fimbriata*, the exogenous application of ABA increased fungal growth slightly. In *Magnaporthe oryzae*, ABA increased spore germination and the formation of appressoria, specialized infection structures for breaking down the plant cell wall and allowing invasion [11]. Moreover, the *Tulasnella* sp. ABA-INSENSITIVE-like protein (Cluster-2641.20009 with 8.94-fold up-regulation) and five ABA receptor PYL-related unigenes (6.54- to 10.06-fold up-regulation) were also found to be significantly up-regulated in the symbiotic germinated seeds compared with the asymbiotic *Tulasnella* sp. fungus. There is also emerging evidence to suggest that ABA plays an important role in compatible interactions in mutualistic plant-microbe interactions [18], and it can promote the infection and establishment of compatible interactions with arbuscular mycorrhizal fungi [16]. However, we found that the ABA concentration in the orchid symbionts is also lower than in the free-living fungal mycelium of *Tulasnella* sp. Based on these results, we can hypothesize that the symbiotic fungus *Tulasnella* produces more activated ABA to promote the recognition and establishment of compatible interactions with its mutualist orchid partner in early seed germination (stages 1–3); it is during these early stages that an abundance of pelotons is usually formed. After protocorm formation, the germinated seed develops to the fourth stage (emergence of leaf from shoot region), the reinvaded hyphae conspicuously decrease, and the hyphae are degraded in the symbiotic orchid cell [34]. Thus, there might be both a collapsed hyphae and healthy pelotons that can be observed. We have detected one putative abscisic acid 8′-hydroxylase-encoding gene, *CYP707A* (Cluster-3911.0), from *Tulasnella* that is highly expressed in symbiotic germinated seeds but not in asymbiotic *Tulasnella*, and this enzyme can degrade and deplete endogenous ABA levels to regulate intracellular ABA homeostasis in plants [16,35]. In line with its important role as a phytohormone, ABA concentrations in the plant are controlled by a tightly regulated balance between biosynthesis, inactivation, and degradation [36]. At this stage, low concentrations of ABA in the symbiont could promote *D. officinale* seed germination and inhibit the growth of *Tulasnella* mycelia.

In our study, we also detected a precursor of SLs (5-DS) in the germinated seeds of *D. officinale*, and in addition, we found that genes encoding key enzymes involved in the conversion of β-carotene to carlactone and mature SLs, *D27*, *CCD7*, *CCD8,* and *MAX1* are expressed in both types of germination seeds, indicating that SLs might also play important roles in regulating *D. officinale* seed germination. Moreover, the content of 5-DS in the asymbiotic germinated seeds (2.03 ng/g·FW) was much higher than in the symbiotic germinated seeds (0.81 ng/g·FW), and this result was supported by the slow growth rate of the asymbiotic germinated seeds compared with that of the symbiotic germinated seeds, indicating that high concentrations of SLs or their biosynthetic precursors might inhibit growth. In other plants, studies have shown that SLs or their biosynthetic precursors act as plant hormones that inhibit shoot branch growth [22,23,24,25]. In addition, the expression of the SL biosynthesis and signal pathway genes increased significantly (>2-fold) in the symbiotic germinated seeds compared to the asymbiotic germinated seeds, which possibly have enhanced the branching of the *Tulasnella* fungus within orchid tissues to maximize the number of pelotons formed. This model might be supported by previous studies showing that SLs also promote branched growth in AM fungi [22,23]. Nevertheless, based on the lower level of 5-DS in the symbiotic germinated seeds and the higher level of 5-DS in the asymbiotic germinated seeds, we can speculate that the symbiotic fungus *Tulasnella* might suppress the biosynthesis of orchid SLs in order to maintain a low concentration of SLs to accelerate the germination rate in the *D. officinale-Tulasnella* symbiosis. Additionally, the germinated seed developed to the fourth stage (emergence of the leaf from the shoot region), the reinvaded hyphae conspicuously decreased, and the hyphae degraded in the symbiotic orchid cell [34]. At this stage, a low concentration of SLs in the symbiont might also slow the growth of the reinvaded *Tulasnella* mycelium.

Previous studies have suggested that ABA is one of the regulators of the strigolactone biosynthesis that acts through an as-yet-unknown mechanism [36]. Moreover, Toh et al. have proposed that host-exudate SLs reduce ABA levels to alleviate seed thermoinhibition in *Arabidopsis* [37,38]. This suggests that there could be complicated cross-talk among the hormones in the orchid-fungus symbionts that warrant future study.

## 4. Materials and Methods

### 4.1. Symbiotic and Asymbiotic Germination of D. officinale Seeds

*D. officinale* plants were grown in the greenhouse of the Chinese Academy of Forestry (Beijing, China) at day/night temperatures of 24 °C/18 °C, a relative air humidity of 65–75%, a 10-h day/14-h night photoperiod, and an irradiance of 700 mol m^−2^·s^−1^. Six *D. officinale* seed capsules were collected from the greenhouse and divided into two groups after surface sterilization. Seeds from the first group were cultured with *Tulasnella* sp., which was isolated from *Cymbidium goeringii* roots (collected from Suizhou, China); a BLAST search showed that the ITS sequences from this isolate were 99% homologous to the ITS from *T. calospora* isolate Pv-PC-2-1 (NCBI accession No: GU166418.1) [39]. The seeds with *Tulasnella* sp. were cultured on 8 g/L oatmeal agar (OMA) medium; this group was defined as DoTc. The second group of seeds was cultured on the same OMA medium without fungi. Seeds were sown and incubated according to the procedures described by Xu et al. [39]. The DoTc seeds germinated and developed to the fourth stage (appearance of the first true leaf, germination rate around at 90%) [12,34], at which point they were cleared in KOH and stained using Trypan blue [39,40]. Microscopic observation showed that the intracellular hyphae stained blue in the DoTc germinated seeds, which indicated that a symbiotic system was established between *D. officinale* and *Tulasnella* sp. The group of seeds germinated without fungi had a very low germination rate (0.1%), and we did not find fungal hyphae; these were defined as asymbiotic germinated seeds (Do). Free-living fungal mycelium was maintained on an OMA medium at 25 °C for 10 days (Tc). DoTc, Do, and Tc (three biological replicates) were collected, immediately frozen in liquid nitrogen, and stored at −80 °C prior to RNA extraction.

### 4.2. cDNA Library Construction and Nucleotide Sequencing

The total RNA was extracted from each sample using the RNeasy Plant Mini Kit (Qiagen, Hilden, Germany) and treated with an RNase-free DNase I digestion kit (Aidlab, Beijing, China) to remove residual genomic DNA. RNA degradation and contamination was monitored by electrophoresis on 1% agarose gels. RNA purity was checked using the NanoPhotometer^®^ spectrophotometer (Implen, Westlake Vilagge, CA, USA). RNA concentration was measured with the Qubit^®^ RNA Assay Kit in the Qubit^®^ 2.0 Fluorometer (Life Technologies, Carlsbad, CA, USA). RNA integrity was assessed using the RNA Nano 6000 Assay Kit on the Bioanalyzer 2100 system (Agilent Technologies, Santa Clara, CA, USA).

For each sample, 3 µg of the total RNA was used as input material for the RNA-Seq library preparations. Sequencing libraries were generated using the NEBNext^®^ Ultra™ RNA Library Prep Kit for Illumina^®^ (NEB, Ipswitch, MA, USA) following the manufacturer’s recommendations, and index codes were added to attribute the sequencing reads to each sample. The PCR products were purified (AMPure XP system) and the library quality was assessed on the Agilent Bioanalyzer 2100. The RNA-Seq libraries were sequenced on an Illumina Hiseq 4000 instrument to generate 150 bp paired-end reads. All samples were sequenced with three biological replicates.

### 4.3. Transcriptome Data Analysis

#### 4.3.1. Quality Control and cDNA Assembly

Raw reads in the FASTQ format were first processed using in-house custom Perl scripts. In this step, clean reads were obtained by removing reads containing adapters, poly-*N*-containing reads, and low quality reads from the raw data. Additionally, the Q20 and Q30 scores, and the GC content of the clean reads were calculated simultaneously. All downstream analyses were based on high-quality clean data.

Sequence read mapping to the *D. officinale* Reference Genome: The index of the *D. officinale* reference genome [27] was built using Bowtie v2.2.3, and the paired-end reads were aligned to the reference genome using TopHat v2.0.12 with mismatch set to 2 and the default values used for the other parameters. The OMF transcriptome de novo assembly was accomplished using Trinity [41] with the min_kmer_cov set to 2 by default and all other parameters set to the default values. The coding sequences were predicted using Estscan v3.0.3 with the default settings [42]. All assembled unigene sequences in this study are given in Table S5. 

#### 4.3.2. Quantification of Gene Expression Levels

HTSeq v0.6.1 was used to count the read numbers mapped to each *D. officinale* gene. The FPKM (expected number of Fragments Per Kilobase of transcript sequence per Million base pairs sequenced) was then calculated for each gene based on the length of the gene and the read count mapped to the gene [43].

Gene expression levels in the OMF were estimated using RSEM with the default settings [44]. The clean reads were mapped to the reference sequences which were assembled by Trinity [41] using the clean read data from the Tc library, and the transcript read counts were obtained and converted to FPKM for gene expression level analysis in the OMF [43].

#### 4.3.3. Differential Gene Expression Analysis

A differential expression analysis of the two conditions/groups (two biological replicates per condition) was performed using the DESeq R package (1.18.0) with the default settings [45]. DESeq provides statistical routines for determining differential expression in digital gene expression data using a model based on the negative binomial distribution. The resulting *p*-values were adjusted using the Benjamini–Hochberg procedure for controlling the false discovery rate. Genes with a log_2_^(Fold_Change)^ > 1 and adjusted *p*-value < 0.001 identified by DESeq were considered to be differentially expressed.

#### 4.3.4. Unigene Functional Annotation and Metabolic Pathway Analysis

Unigene function was annotated based on the seven databases reported by Liu et al. [46]. Metabolic pathway analysis was performed using the Kyoto Encyclopedia of Genes and Genomes Pathway database (KEGG: http://www.genome.jp/kegg/). We used the KOBAS [47] software to test the statistical enrichment of DEGs in the KEGG pathways.

### 4.4. Hormone Extraction, Fractionation, and UPLC-ESI-qMS/MS Analysis

The concentrations of the ABA, JA, and SL hormones in the Do, DoTc, and Tc samples were measured using ultraperformance liquid chromatography coupled with a tandem quadrupole mass spectrometer equipped with an electrospray interface (UPLC-ESI-qMS/MS). Six biological replicates were used in the hormone quantification measurements.

1. 5-DS: To isolate 5-DS, 1 g (fresh weight) samples of symbiotic or asymbiotic seeds were frozen in liquid nitrogen, ground to a powder, transferred to 2 mL microcentrifuge tubes, and then extracted with 10 mL acetone with ultrasound in a water bath for 15 min, after which the tubes were kept at −20 °C for at least 12 h. After centrifugation at 8000 rpm for 5 min, the supernatants were transferred into new tubes and centrifuged at 13,000 rpm at 4 °C for 5 min. The lower organic phases were then transferred to new tubes and evaporated to dryness in a stream of dry N_2_. The crude extracts were dissolved in 3 mL normal hexane and filtered through spin columns (500 mg, 6 mL, Agilent Technologies Corporation, Santa Clara, CA, USA) with 5 mL of normal hexane, and the elution products were then evaporated to dryness in a stream of N_2_. The crude extracts were then dissolved in 200 μL of methanol and filtered through spin columns (Ultra-Free MC, 0.22 µm pore size; Millipore, Tokyo, Japan), and subjected to an HPLC-MS/MS analysis.

Identification of 5-DS by LC–MS/MS: HPLC separation was conducted with an Agilent 1290 HPLC instrument (Agilent Technologies Corporation) fitted with a poroshell 120 SB-C18 column (2.1 mm × 150 mm, 2.7 μm), and 2 µL was injected for the HPLC-MS/MS analysis. The column temperature was kept at 30 °C. The binary mobile phase system consisted of methanol (A) and water (B) in 0.1% formic acid under a gradient condition (0–0.5 and 5.1–8.00 min, 80% A, 3–5 min, 90% B) with a flow rate of 0.3 mL/min. Mass spectrometry was performed with a SCIEX-6500Qtrap (MS/MS) mass spectrometer (AB SCIEX LLC., Framingham, MA, USA) equipped with an electrospray source. The drying and nebulizing gas was nitrogen generated from pressurized air in an N2G nitrogen generator (MIU instruments, Hangzhou, China). The curtain gas was set to 25 psi, the nebulizer gas was set to 55 psi, the heater gas was set to 65 psi, and the ion spray voltage was set to 5000 V. The atomization temperature was set to 350 °C. Analytes were quantified in the multiple-reaction-monitoring (MRM) mode for the known 5-DS detection, at *m*/*z* 331.0/234.1 with a declustering potential (DP) of 45 V and collision energy (CE) of 14 V.

2. ABA and JA: To isolate the hormones, 1 g (fresh weight) samples of symbiotic or asymbiotic seeds were frozen in liquid nitrogen, ground to a powder, and transferred to 2 mL microcentrifuge tubes. The ground tissue was extracted in 10 mL isopropanol/hydrochloric acid buffer by shaking at 4 °C for 30 min, and 20 mL of dichloromethane was then added and the mixture was vibrated at 4 °C for a further 30 min. The tubes were centrifuged at 13,000 rpm at 4 °C for 5 min, and the lower organic phase was transferred to a new tube and was then evaporated to dryness in a stream of N_2_. The extracts were dissolved in 400 μL of methanol and filtered through spin columns (Ultra-Free MC, 0.22 µm pore size; Millipore, Tokyo, Japan), and subjected to an HPLC-MS/MS analysis.

ABA and JA were measured with an HPLC-ESI-qMS/MS instrument (AQUITY UPLC™ System/Quattro Ultima Pt; Waters, Milford, MA, USA) fitted with an AQUITY UPLC BEH C18 column (1.7 µm, 2.1 × 100 mm, Waters, Milford, MA, USA). The column temperature was kept at 40 °C. A binary mobile phase system consisting of 2% (*v*/*v*) methanol, 0.05% formic acid, and 5 mmol/L ammonium acetate in water (A) and acetonitrile (B) under a gradient condition (0–0.25 and 5.01–6.00 min, 90% (A), 4–5 min, 100% (B)) with a flow rate of 0.3 mL/min. The sample chamber temperature was set to 10 °C and the injection volume was 10 μL. The capillary voltage was controlled at 20 V. The ion source temperature was set to 150 °C, the cone gas flow was 50 L/h, desolvation temperature was 400 °C, and the desolvation gas flow was 800 L/h. Analytes were also quantified in multiple-reaction-monitoring (MRM) mode for the known ABA and JA detections.

For ABA quantification, the declustering potential was −25 V, the collision energy −25 V, and the ion transition was at *m*/*z* 263.2/204.0; for JA quantification, the declustering potential was −32 V, the collision energy −12 V, and the ion transition was at *m*/*z* 59.0/109.0.

### 4.5. Differential Gene Expression Detected by qRT-PCR

The symbiotic and asymbiotic root samples from the *D. officinale* seedlings (10 cm in height) that were previously used to prepare the RNA-Seq libraries were used to perform qRT-PCR assays for gene expression quantification. The plant materials were flash-frozen in liquid nitrogen and stored at −80 °C prior to RNA extraction. RNA isolation, qRT-PCR, and calculations of relative mRNA levels were performed as previously described [48]. Each data point represents the mean of three biological replicates and three experimental replicates. *DoEF1α* and *DoRPL30* [49] from *D. officinale*, and *TcEF1α* [50] and *TcTUBB* (Cluster-2641.19171, Table S5) from *Tulasnella* were used as reference genes for the normalization of gene expression, respectively. The primer sequences are shown in Table S9, and their amplification efficiencies were within the range of 95–105%.

## 5. Conclusions

In this study, we generated transcriptome datasets of *D. officinale* germinated seeds to examine the expression profiles of genes related to the biosynthesis of three types of endogenous hormones—JA, ABA, and SLs—in the *D. officinale-Tulasnella* symbionts, asymbiotic germinated seeds, and the free-living OMF. We also performed comprehensive analyses of the contents of individual hormones, and assayed gene expression using qRT-PCR in the symbiotic and asymbiotic seed samples. The results allowed us to propose potential regulatory mechanisms for JA, ABA, and SLs from the orchid and OMF in the *D. officinale-Tulasnella* symbionts. Our study will provide new insights into the molecular mechanisms that regulate the orchid-fungus symbiosis.

## Figures and Tables

**Table 1:** The DEGs in the DoTc vs. Do and DoTc vs. Tc comparisons involved in the ABA (abscisic acid), JA (jasmonic acid), and SL (strigolactone) biosynthesis pathways.

Gene Name	Gene ID		Description
ABA pathway		log2(DoTc/Do)	
*DoZXE-1*	Dendrobium_GLEAN_10076665	−2.11	Zeaxanthin epoxidase
*DoZXE-2*	Novel00252	−2.00	Zeaxanthin epoxidase
*DoSDR-1*	Dendrobium_GLEAN_10042288	−0.57	Short-chain type dehydrogenase/reductase
*DoSDR-2*	Dendrobium_GLEAN_10068100	−1.33	Short-chain type dehydrogenase/reductase
*DoSDR-3*	Dendrobium_GLEAN_10127951	−2.1	Short-chain type dehydrogenase/reductase
ABA pathway		log2(DoTc/Tc)	
*BcABA4-1*	Cluster-2641.12008	7.24	Short-chain type dehydrogenase/reductase dehydrogenase/reductase
*BcABA4-2*	Cluster-2152.0	2.42	Short-chain type dehydrogenase/reductase
*BcABA1/2-1*	Cluster-2641.19250	10.43	P450 monooxygenase protein CYP51
*BcABA1/2-2*	Cluster-2641.15199	10.01	P450 monooxygenase protein CYP51
JA pathway		log2(DoTc/Do)	
*DoLOX*	Dendrobium_GLEAN_10090503	−0.67	Linoleate 13S-lipoxygenase
*DoAOS-1*	Dendrobium_GLEAN_10010006	−0.98	Allene oxide synthase
*DoAOS-2*	Dendrobium_GLEAN_10012197	−0.40	Allene oxide synthase
*DoOPR*	Dendrobium_GLEAN_10113554	−1.65	12-oxophytodienoate reductase
*DoKAT*	Dendrobium_GLEAN_10097185	−2.50	3-ketoacyl-CoA thiolase
JA pathway		log2(DoTc/Tc)	
*TcLOX-1*	Cluster-2641.17948	10.60	Linoleate 13S-lipoxygenase
*TcLOX-2*	Cluster-2641.19486	10.33	Linoleate 13S-lipoxygenase
*TcLOX-3*	Cluster-2641.18695	10.34	Linoleate 13S-lipoxygenase
*TcLOX-4*	Cluster-2641.17988	10.66	Linoleate 13S-lipoxygenase
*TcLOX-5*	Cluster-2641.16009	9.35	Linoleate 13S-lipoxygenase
*TcLOX-6*	Cluster-6210.0	6.67	Linoleate 13S-lipoxygenase
*TcLOX-7*	Cluster-1378.1	5.99	Linoleate 13S-lipoxygenase
*TcAOS-1*	Cluster-2641.19289	10.40	Allene oxide synthase
*TcAOS-2*	Cluster-2641.20726	10.07	Allene oxide synthase
*TcAOS-3*	Cluster-2641.16940	7.81	Allene oxide synthase
*TcAOS-4*	Cluster-2641.8055	6.54	Allene oxide synthase
*TcAOS-5*	Cluster-2641.30606	7.21	Allene oxide synthase
SL pathway		log2(DoTc/Do)	
*DoHMGR-1*	Dendrobium_GLEAN_10079864	0.87	Hydroxymethylglutaryl-CoA reductase
*DoHMGR-2*	Dendrobium_GLEAN_10079865	0.91	Hydroxymethylglutaryl-CoA reductase
*DoD27*	Dendrobium_GLEAN_10126990	5.22	β-carotene isomerase
*DoCCD7-1*	Dendrobium_GLEAN_10008301	4.21	Carotenoid cleavage dioxygenase 7
*DoCCD7-2*	Dendrobium_GLEAN_10009315	2.98	Carotenoid cleavage dioxygenase 7
*DoCCD7-3*	Dendrobium_GLEAN_10049685	9.92	Carotenoid cleavage dioxygenase 7
*DoCCD8-1*	Dendrobium_GLEAN_10070249	3.87	Carotenoid cleavage dioxygenase 8
*DoCCD8-2*	Dendrobium_GLEAN_10072088	4.08	Carotenoid cleavage dioxygenase 8
*DoMAX1*	Dendrobium_GLEAN_10109899	2.00	Cytochrome P450 711A1
*DoD14-1*	Dendrobium_GLEAN_10025168	2.45	Probable strigolactone esterase
*DoD14-2*	Dendrobium_GLEAN_10114182	1.52	Probable strigolactone esterase
*DoD14-3*	Dendrobium_GLEAN_10143398	1.86	Probable strigolactone esterase
*DoDAD2-1*	Dendrobium_GLEAN_10013491	2.01	Probable strigolactone esterase
*DoDAD2-2*	Dendrobium_GLEAN_10114181	1.34	Probable strigolactone esterase

## Supplementary Materials

The following are available online at http://www.mdpi.com/1422-0067/19/11/3484/s1.
