# Supplementary material for: Functional Insights into the Roles of Hormones in the Dendrobium officinale-Tulasnella sp. Germinated Seed Symbiotic Association
Source: Int J Mol Sci. 2018 Nov 6;19(11):3484. doi: 10.3390/ijms19113484 (PMC6274778; doi:10.3390/ijms19113484)
Supplement: Supplementary file 1 [file ijms-19-03484-s001.zip › ijms-376543-supplementary file/Supplementals/Figure S2.pdf]

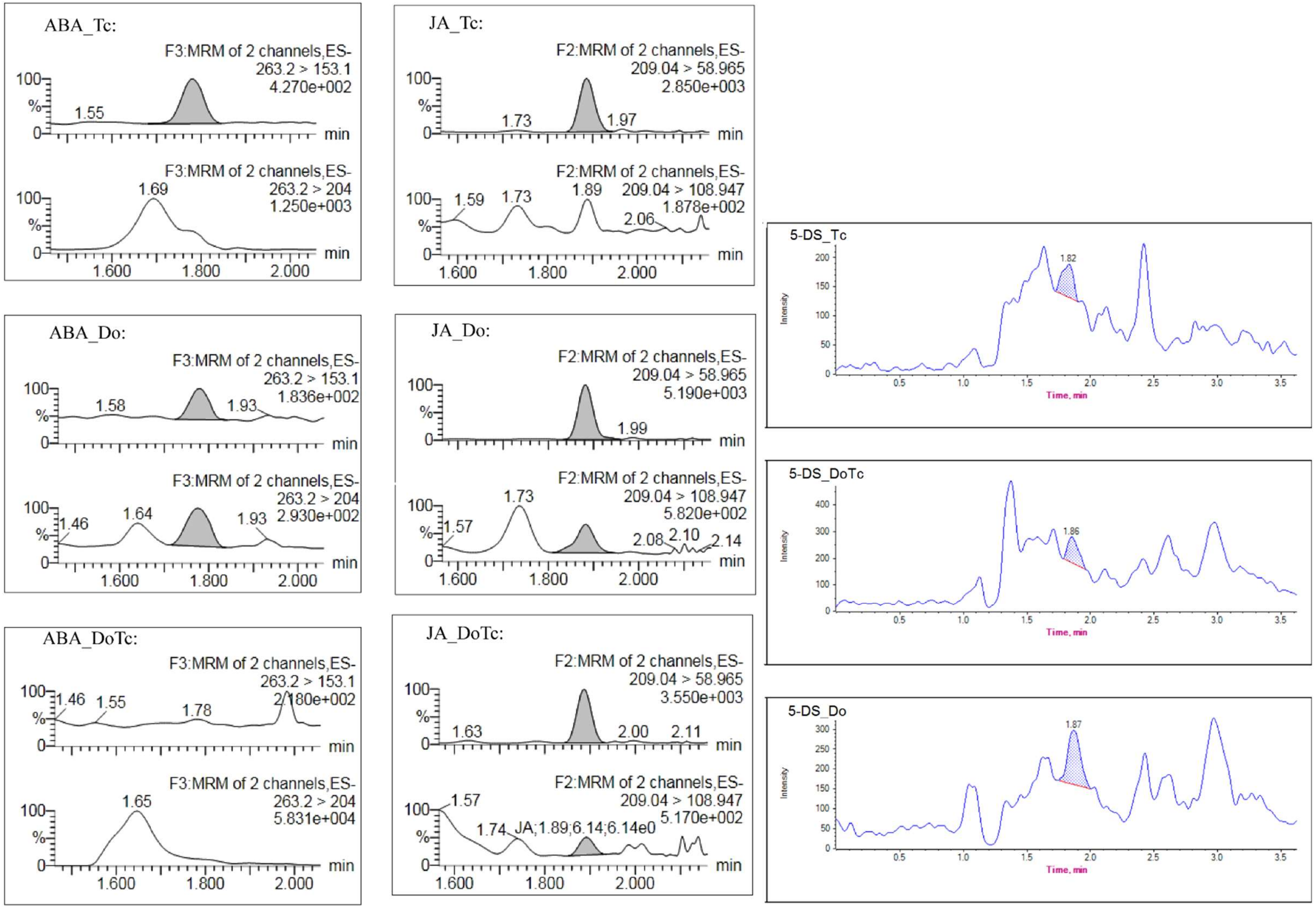

Figure S2. The UPLC-ESI-qMS/MS spectra detection results of ABA, JA and 5-DS in the symbiotic germinated seeds (DoTc), asymbiotic germinated seeds (Do) and free-living OMF *Tulasnella* sp. (Tc).
