# Supplementary figures and images for: Functional Insights into the Roles of Hormones in the Dendrobium officinale-Tulasnella sp. Germinated Seed Symbiotic Association
Source: Int J Mol Sci. 2018 Nov 6;19(11):3484. doi: 10.3390/ijms19113484 (PMC6274778; doi:10.3390/ijms19113484)

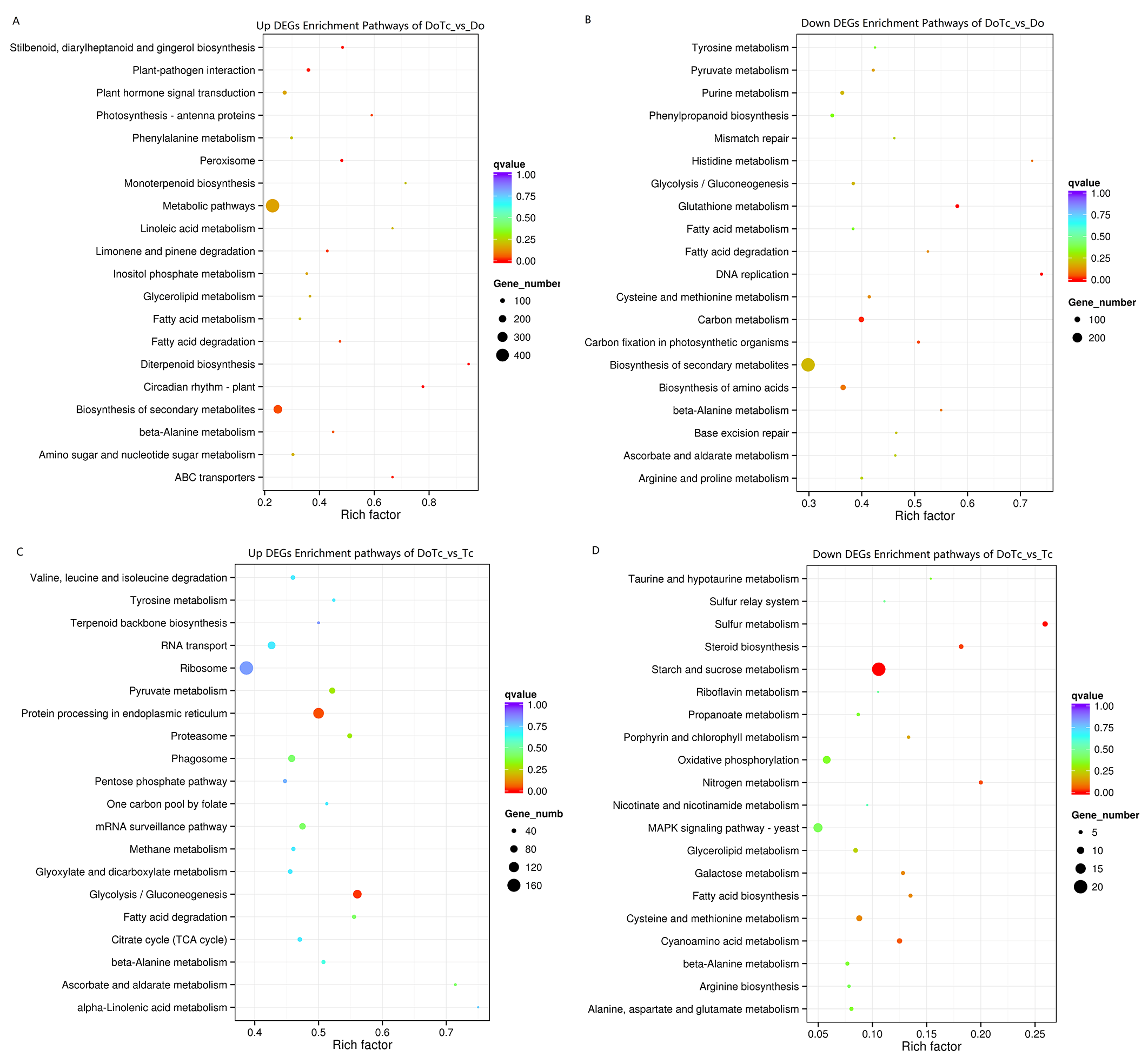

Supplement: Supplementary file 1 [file ijms-19-03484-s001.zip › ijms-376543-supplementary file/Supplementals/Figure S1.tif]
